# Progress in Medicine

**Published:** 1900-08-18

**Authors:** 


### Progress in Medicine.

### NEUROLOGY.

(*Continued from page* 319.)

**Amaurotic Family Idiocy** was first described by Warren Tay in 1881, and subsequently by Sachs and Hirsch. The principal features are briefly as follows: Semitic parentage, with no evidence of inherited syphilis or other diathesis; nearly always more than one child in the same family attacked by the disease; normal birth at full term and normal development for a few months, followed by rather rapid physical and mental failure, together with loss of vision and peculiar fundus changes, the child dying of marasmus at about the age of two years. There may or may not be nystagmus, strabismus, rigidity, or contractures, and the reflexes may be diminished or exaggerated. In a large proportion of the patients abnormal sensitiveness to noise is present, or at least an exaggerated reaction to auditory impressions. Two such cases have recently been described by Patrick[5] and by Kuh.[6] The first corresponded to the type described above, except that the parents were not Jews and not blood relations. In the second the peculiar features were the great frequency of epileptic spasms, and the existence of a pronounced hydrocephalus. The ocular fundi in each case were examined by Beard,[7] who said that they were exactly similar and typical of the disease. The optic disk is of normal size, with some blanching of its outer half. The dominating feature of the picture, which is absolutely diagnostic of the disease in question, is that surrounding the fovea centralis and concentric with it, though two or three times as large, is a liver-coloured circle. This circle is the centre of a zone of greyish-white which gradually fades away into the normal red-orange of the retina. This livid disk is clear-cut, not irregular in outline as in acute inflammatory conditions, nor cherry-red, but distinctly brownish. The retinal appearance thus bears out the assertion of Holden that the disease is attended by degeneration of the ganglion cells of the retina, which, as is well known, are piled one on another at the rim of the fovea, and gradually thin down as the distance from the centre increases.

**Gastric Crises of Tabes.**—Basch[8] contributes an important study of 25 cases of gastric crises of tabes. The majority of the patients were between 30 and 40 years of age; no history of heredity of the crises could be obtained. Most patients said that their attacks occurred suddenly and without any assignable cause. They had lasted in some cases for 15 years. The length of the single attack varied within wide margins, even in the same case—from 10 to 24 hours up to three to six weeks, the majority lasting from one to seven days. The interval was also very variable. If the crises occurred in the early stage of tabes, the general condition during the interval was good, when occurring frequently and with great severity there was developed a condition similar to chronic dyspepsia or ulcer of the stomach. In all cases vomiting took place; and in all but three pain was marked, and formed the most prominent symptom of the entire attack, it was as a rule either in the epigastrium or back. In the majority the vomiting ameliorated the severity of the pain. The quantity of vomited matter was, as a rule, enormous, the vomit consisting at first of food and finally of a tasteless, clear mucus, or of bile-coloured fluid, in one case coffee-ground vomiting occurred. The vomited matter did not contain any large amount of hydrochloric acid. In no case did palpation or percussion of the abdomen produce any pain whatever. This is of considerable importance from the point of view of diagnosis between nervous vomiting and that which accompanies acute inflammatory conditions of the abdominal organs. In one case attacks of syncope complicated the crises. A convenient classification of gastric crises is into those with rapid and those with gradual onset, and those with marked prominence of isolated manifestations, *e.g.*, "anorexie tabétique" (Fournier), "variété flatulante" (Fournier), "vomissements noirs" (Charcot). Typical crises have the following well-defined characteristics: (1) absence of symptoms of organic disease of the gastro-intestinal tract; (2) sudden onset; (3) no initial chill; (4) great intensity of symptoms, sometimes rapidly, sometimes gradually increasing in severity; (5) rapid pulse with no fever; (6) absence of specific diagnostic features in the vomit; (7) external circumstances have no influence upon the natural course of the attack; (8) spontaneous cessation; (9) rapid convalescence.

The diseases with which tabetic gastric crises are most likely to be confounded are ulcer and cancer of the stomach, duodenal ulcer, gall stones, cerebral vomiting, and the vomiting of uræmia. The positive diagnosis of genuine gastric crises rests naturally on the diagnosis of locomotor ataxy itself. In gastric ulcer the vomit always contains large quantities of free hydrochloric acid, and it may be blood, and there is great tenderness on palpation. In duodenal ulcer the fæces may contain blood, and the vomiting does not continue for days as in tabetic crises. Mistakes can only arise in atypical attacks of gall stone colic, *i.e.*, those unaccompanied by jaundice. Pure cerebral vomiting resembles tabetic crises in that it occurs periodically, independently of food, and may continue day and night, but it is not preceded by nausea, and is not accompanied by abdominal pain.

The one sovereign remedy during the attack is hypodermic injection of morphia. Oxalate of cerium, antipyrin, or strychnine nitrate may be tried as substitutes, as they do good in exceptional cases. The second indication—to provide food during the attack—may become a very earnest question, if the attacks are prolonged and the patient poorly nourished. A fluid diet, beginning with teaspoonful doses every half-hour, should be tried. In very prolonged cases rectal alimentation is necessary. Convalescence is marked by a very strong appetite, and it is generally necessary to warn patients against over-eating. And during the intervals one must try to raise and keep up the general nutritive standard, for there are no therapeutic measures known which will cause either disappearance of gastric crises or a diminution in their frequency.

**The Status Epilepticus**[9] is now well recognised as the acme or climax of epilepsy. The essentials of the condition are rapidly-repeated seizures and progressive deepening coma. There is also a marked elevation in the fever, pulse, and respiratory curves. Recent investigations show that there is a necessity for reviving the old theory of cortical heat centres, for though the fever curve is generally in direct ratio to the severity and number of epileptic convulsions, this rule is not always true, so that the fever is not altogether due to the muscular contractions. In the majority of cases the disease of epilepsy must be very well established before status can supervene. But few patients recover from their disease when status has once occurred. The immediate cause of status is still as indefinite as that of epilepsy proper, and the onset of status does not differ from the beginning of serial attacks. The advance of our knowledge of the treatment of status epilepticus is not great. The percentage of recoveries remains about 60 or 70, in spite of improved methods of treatment. It should be borne in mind that what is indicated in the convulsive stage is contra-indicated in the stuporous stage, and the administration of large doses of chloral, uncombined with other drugs, has come to be considered as dangerous in the light of its depressing effects upon the heart. In spite of frequent abscess formations from hypodermic bromide medication, this line of treatment is steadily growing in favour in the severest cases of status.

**Effect of Alcohol on the Brain.**—Horsley,[10] in the Lees and Raper memorial lecture, said that the simple reaction period, the mere response to a signal after the ingestion of a small quantity of alcohol, was slightly quickened; in the course of a few minutes, however, a slowing began and persisted as long as the alcohol was in active operation in the body. In complex reactions even small quantities of alcohol slowed the time limit from the very first. In the performance of muscular work the amount done was at first increased, and then diminished. The cerebellum, which is especially concerned in equilibration, is also affected by alcohol, so that the individual staggered slightly and found standing a matter of difficulty. The cerebellum also has the function of damping down the tremor which normally accompanies the discharge of energy from a nerve centre. Hence it is possible that alcoholic tremor may be due in part to loss of cerebellar controlling influence.

Chronic alcoholism produces degeneration of the cell-structure of Purkinjee's cells, and alteration of the dendrites of the cortical pyramidal cells, with widespread pigmentation.

**Narcolepsy** is a disease characterised as a peculiar neurosis, characterised by an irresistible desire to sleep, occurring suddenly, of short duration, and recurring at frequent intervals. These recurring attacks are considered by some authors to belong to epilepsy, by others to be due to visceral derangement; by others, again, to be merely a symptom-complex prominently associated with the other phenomena of degeneration. McCarthy,[1[illegible]] who holds the last opinion, cites four cases illustrating the differences in the clinical pictures in different cases. The first case was that of a young coloured woman, 19 years of age, who for five years had been having sudden attacks of morbid sleep at irregular intervals. The discovery of slight but wide-spread sensory changes stamped the case as one of hysteria, and excluded the possibility of its being one of true narcolepsy. The second case was that of a white woman, 42 years of age, who had several attacks of prolonged sleep lasting from three days to a week each, and also an attack of hysteria major. Besides the hysteria in this case there was neurasthenia, and possibly a beginning melancholia, as factors in the production of sleep.

Hysterical conditions should be differentiated as a class from the other conditions of the organism which produce morbid sleep. The latter may be divided into two classes: (1) The epileptoid sleeping states, such as petit mal, epileptic equivalent, &c.; and (2) the morbid sleep depending on toxæmic states, and disturbed cerebral circulation and nutrition; to this class belong the somnolence of uræmia, cholæmia; of pulmonary and cardiac diseases; of organic brain disease; of toxic products from without such as opium; and the cerebral exhaustion of typhoid and some blood diseases.

[5] Patrick, Jour. of Nerv. and Med. Dis., May, 1900. [6] Kuh, Jour. of Nerv. and Med. Dis., May, 1900. [7] Beard, Jour. of Nerv. and Med. Dis., May, 1900. [8] Basch, Med. Rec., Oct. 14, 1899. [9] Med. Rec., Mar. 3, 1900. [10] Horsley, Lancet, May 5, 1900. [11] McCarthy, Amer. Jour. Med. Sci., Feb., 1900.

